# Public Health Threat of New, Reemerging, and Neglected Zoonoses in the Industrialized World

**DOI:** 10.3201/eid1601.081467

**Published:** 2010-01

**Authors:** Sally J. Cutler, Anthony R. Fooks, Wim H. M. van der Poel

**Affiliations:** University of East London, London, UK (S.J. Cutler); Veterinary Laboratories Agency, Addlestone, UK (A.R. Fooks); University of Liverpool, Liverpool, UK (A. R. Fooks, W.H.M. van der Poel); Central Veterinary Institute of Wageningen University and Research Centre, Lelystad, the Netherlands (W.H.M. van der Poel)

**Keywords:** Zoonoses, bacteria, viruses, parasites, infectious diseases, arthropod-borne disease, new zoonoses, emerging diseases, reemerging infections, synopsis

## Abstract

Microbiologic infections acquired from animals, known as zoonoses, pose a risk to public health. An estimated 60% of emerging human pathogens are zoonotic. Of these pathogens, >71% have wildlife origins. These pathogens can switch hosts by acquiring new genetic combinations that have altered pathogenic potential or by changes in behavior or socioeconomic, environmental, or ecologic characteristics of the hosts. We discuss causal factors that influence the dynamics associated with emergence or reemergence of zoonoses, particularly in the industrialized world, and highlight selected examples to provide a comprehensive view of their range and diversity.

## Abstract 2

Improving our capacity to respond to these pathogens is essential.

## CME ACTIVITY

MedscapeCME is pleased to provide online continuing medical education (CME) for this journal article, allowing clinicians the opportunity to earn CME credit. This activity has been planned and implemented in accordance with the Essential Areas and policies of the Accreditation Council for Continuing Medical Education through the joint sponsorship of MedscapeCME and Emerging Infectious Diseases. MedscapeCME is accredited by the Accreditation Council for Continuing Medical Education (ACCME) to provide continuing medical education for physicians. MedscapeCME designates this educational activity for a maximum of 0.5 *AMA PRA Category 1 Credits*™. Physicians should only claim credit commensurate with the extent of their participation in the activity. All other clinicians completing this activity will be issued a certificate of participation. To participate in this journal CME activity: (1) review the learning objectives and author disclosures; (2) study the education content; (3) take the post-test and/or complete the evaluation at **http://www.medscape.com/cme/eid**; (4) view/print certificate.

## Learning Objectives

Upon completion of this activity, participants will be able to:

- List animal hosts for different zoonoses
- Identify the type of zoonosis most likely to undergo genetic mutation
- Specify factors that increase the risk for zoonoses now and in the future.

## Editor

**Thomas Gryczan**, Technical Writer-Editor, *Emerging Infectious Diseases. Disclosure: Thomas Gryczan has disclosed no relevant financial relationships*.

## CME AUTHOR

**Charles P. Vega, MD,** Associate Professor; Residency Director, Department of Family Medicine, University of California, Irvine. *Disclosure: Charles P. Vega, MD, has disclosed no relevant financial relationships.*

## AUTHORS

Disclosures: **Sally J. Cutler, PhD**, has disclosed the following relevant financial relationship: served as an advisor to the Institute of Biomedical Sciences virology panel. **Anthony R. Fooks, PhD, CBiol, FiBiol, BSc (Hons)**, has disclosed the following relevant financial relationships: received grants for educational activities from The Wellcome Trust, World Health Organization, University of Oxford Medical Research Fund, UK Health Protection Agency, and UK Department for Environment, Food and Rural Affairs; served as an advisor or consultant for World Health Organization, World Health Organization for Animal Health, UK Department for Environment, Food and Rural Affairs. **Wim H.M. van der Poel, PhD, DVM**, has disclosed no relevant financial relationships.

## Earning CME Credit

To obtain credit, you should first read the journal article. After reading the article, you should be able to answer the following, related, multiple-choice questions. To complete the questions and earn continuing medical education (CME) credit, please go to **http://www.medscape.com/cme/eid**. Credit cannot be obtained for tests completed on paper, although you may use the worksheet below to keep a record of your answers. You must be a registered user on Medscape.com. If you are not registered on Medscape.com, please click on the New Users: Free Registration link on the left hand side of the website to register. Only one answer is correct for each question. Once you successfully answer all post-test questions you will be able to view and/or print your certificate. For questions regarding the content of this activity, contact the accredited provider, CME@medscape.net. For technical assistance, contact CME@webmd.net. American Medical Association’s Physician’s Recognition Award (AMA PRA) credits are accepted in the US as evidence of participation in CME activities. For further information on this award, please refer to http://www.ama-assn.org/ama/pub/category/2922.html. The AMA has determined that physicians not licensed in the US who participate in this CME activity are eligible for *AMA PRA Category 1 Credits*™. Through agreements that the AMA has made with agencies in some countries, AMA PRA credit is acceptable as evidence of participation in CME activities. If you are not licensed in the US and want to obtain an AMA PRA CME credit, please complete the questions online, print the certificate and present it to your national medical association.

### Article Title: Public Health Threat of New, Reemerging, and Neglected Zoonoses in the Industrialized World

## CME Questions

1. Which of the following infections is correctly matched with its animal host?
  A. Q fever → fish, domestic animals, birds, and ticks
  B. Rift Valley fever → pigeons
  C. Leishmaniasis → chimpanzees
  D. *Bartonella henselae* → dogs
2. Which of the following organisms is most likely to mutate?
  A. Yeasts
  B. Fungi
  C. Bacteria
  D. Viruses
3. Which of the following statements about emerging zoonotic infections is most accurate?
  A. Water sports can expose humans to brucellosis
  B. Importation of animals for sport does not affect rates of infection
  C. The greatest risk for human rabies comes from dogs
  D. Of all bushmeat, nonhuman primates afford the lowest risk for infection
4. Which of the following statements about rickettsial disease is most accurate?
  A. Increased tourism has not influenced the prevalence of rickettsial disease
  B. Neuropathy is evident within 3 months of infection
  C. Spotted fevers are an emerging zoonosis
  D. Rates of Lyme borreliosis in humans have fallen

### Activity Evaluation

**Table:** 

**1. The activity supported the learning objectives.**				
Strongly Disagree				Strongly Agree
1	2	3	4	5
**2. The material was organized clearly for learning to occur.**				
Strongly Disagree				Strongly Agree
1	2	3	4	5
**3. The content learned from this activity will impact my practice.**				
Strongly Disagree				Strongly Agree
1	2	3	4	5
**4. The activity was presented objectively and free of commercial bias.**				
Strongly Disagree				Strongly Agree
1	2	3	4	5

## Public Health Threat of New, Reemerging, and Neglected Zoonoses in the Industrialized World

The World Health Organization/Food and Agriculture Organization/World Organisation for Animal Health joint consultation on emerging zoonotic diseases, held in Geneva in 2004, defined an emerging zoonosis as “a pathogen that is newly recognized or newly evolved, or that has occurred previously but shows an increase in incidence or expansion in geographical, host or vector range” (www.who.int/zoonoses/emerging_zoonoses/en). Through continued alterations in human and animal demographics and environmental changes, new and recurring diseases are likely to continue to emerge.

The effects of zoonoses on human health and economics have recently been underscored by notable outbreaks such as those involving Nipah virus and severe acute respiratory syndrome (SARS) coronavirus (CoV). A recent retrospective study of 335 emerging infectious episodes over a 64-year period (1940–2004) emphasized the role of wildlife as a source of emerging infections. However, research efforts have typically been focused toward either humans or economically related species (*1*).

The frequency of these events increased substantially over the period of investigation (*2*). Such infections are now often perceived as agents of biologic warfare rather than infections with a long but insidious history in their appropriate ecologic niche. Why then are these infections becoming a serious public health concern? The answer is a complex multifactorial set of changing circumstances. To support the growing human population, we have an increasing demand for nutritional support, resulting in intensive agricultural practices, sometimes involving enormous numbers of animals, or multiple species farmed within the same region. These practices can facilitate infection to cross species barriers.

Additionally, we are witnessing increasing globalization, with persons (*3*), animals, and their products (*4*) moving around the world. This movement enables unprecedented spread of infections at speeds that challenge the most stringent control mechanisms. Furthermore, continual encroachment of humans into natural habitats by population expansion or tourism brings humans into new ecologic environments and provides opportunity for novel zoonotic exposure. Climatic changes have facilitated the expansion of compatible conditions for some disease vectors, remodeling dynamics for potentially new, emerging, and reemerging zoonoses (*5*). In the next 2 decades, climate change will be the most serious issue that dominates reemergence of pathogens into new regions.

Climate change also effects evolution of pathogens, and where relevant, their vectors. Continual mutation and recombination events give rise to variants with altered levels of fitness to persist and spread. Changing ecologic circumstances and pathogen diversity can give rise to variants with altered pathogenic potential. However, the host must not be ignored. Increased longevity and therapies for persons with diseases can modulate host susceptibility and concomitant infections and upset the evolving and dynamic infection balance.

## Emerging, Reemerging, and Neglected Zoonoses

Data for this review were identified in PubMed searches and relevant journal articles and excluded those studies not published in English. Emerging or reemerging pathogens must be considered on multiple levels. First, pathogens not previously known have been identified. For example, alteration in the processing of cattle feed in the United Kingdom resulted in extended host range and emergence of bovine spongiform encephalopathy in cattle (*6*). Similarly, mixing of multiple species under stressful conditions can promote a species jump such as that witnessed with SARS-CoV (*7*). New opportunities can be created by climatic changes such as global warming and ecologic alterations facilitated through changed land use and movements of infected hosts, susceptible animals, or disease vectors.

In 1987, 1997–1998, and 2006–2007, outbreaks of infection with Rift Valley fever virus in Africa were associated with changes in river flow and flooding resulting from damming of rivers or heavy rainfall. Many zoonotic pathogens fall into the category of generalist agents exhibiting extensive host diversity, e.g., *Coxiella burnetii*, the etiologic agent of Q fever. This bacterium can successfully infect hosts ranging from domestic animals to wildlife, reptiles, fish, birds, and ticks.

Others agents have restricted specific transmission dynamics because of limited host ranges. These agents include simian immunodeficiency viruses 1 and 2, which are found in chimpanzees and sooty mangabees, and Rift Valley virus, which is transmitted by *Aedes* spp. and *Culex* spp. mosquitoes and found in sheep and goats. For many zoonotic agents, the potential to cause infection in accidental hosts, such as humans, exists, but often this represents a dead-end host. Pathogens such as *Anaplasma* spp., *Erhlichia* spp., *Rickettsia* spp., *Bartonella* spp., West Nile virus, and rabies virus can be included in this group.

From an epidemiologic point of view, “A reservoir should be defined as one or more epidemiologically connected populations or environments in which a pathogen can be permanently maintained and from which infection is transmitted to the defined target species” (*8*). Conversely, some zoonoses in specific conditions show remarkable ability for human-to-human transmission beyond the confines of natural sylvatic cycles. This ability was seen during a recent outbreak of plague among diamond miners in the Congo. This outbreak was initiated by an infection of a miner, which became pneumonic and resulted in 136 secondary cases of pneumonic plague and 57 deaths (*9*). Transmission of plague is complex and dynamic, with combinations of stochastic and adaptive mechanisms. As seen in this example, rapid transmission often occurs, but this is accompanied by slower, localized transmission among enzootic reservoir species, which often use vector-borne expansion among low-density hosts (*10*). Other zoonoses, given correct circumstances, can result in human-to-human transmission. These zoonoses include those that cause Ebola fever, influenza A, plague, tularemia, and SARS (*11*).

New or emerging virulence traits can evolve and result in large-scale transmission and concomitant alteration of pathogenicity. This new pathogenicity may include increased invasiveness, enhanced spread, toxin production, or antimicrobial drug resistance. *Y*. *pestis* has shown a resurgence in regions such as Madagascar, with isolates showing a marked increase in resistance to antimicrobial agents (*12*). Similarly, a recently evolved outer surface protein A serotype of a Lyme borreliosis spirochete (*Borrelia garinii* serotype 4), has shown particularly aggressive tendencies and is often associated with hyperinvasive infection (*13*). Concern has also been noted about increasingly frequent isolation of *Corynebacterium ulcerans* carrying the diphtheria toxigenic phage.

Mutation is the ultimate source of genetic variation, on which natural selection, genetic drift, gene flow, and recombination act to shape the genetic structure of populations. This factor is especially notable in viruses, which have relatively small genomes and short generation times, particularly among viruses with more error-prone RNA genomic replication (*14*). However, most mutations are deleterious and under pressure of innate and adaptive host immunity, viruses probably always experience selection for mutation rates >0. The upper limit on mutation rates will be determined by factors such as natural selection, genomic architecture, and the ability to avoid loss of viability or genetic information, albeit, that a loss of genetic information and increased specialization is observed in co-evolution with a host (*15*).

According to evolutionary theory, higher mutation rates should be favored in a changing environment, such as altered host immune defenses. However, in experimental settings, artificially increased mutation rates are often associated with lower virus titers. In addition, a complex relationship exists between underlying mutational dynamics and the ability to generate antigenic variation, which in turn has serious implications for the epidemiologic potential of the virus.

Evolutionary changes are not always a prerequisite for viral emergence in a new host. Some viruses (e.g., poxviruses), have a wide host range and show a relatively low mutation rate. However, in other viruses such as Venezuelan equine encephalitis virus, evolutionary change is essential for efficient infection and transmission to new hosts (*16*). Because most viruses replicate poorly when transferred to new hosts, greater variation is more likely to assist viral adaptation to its new host.

All too frequently, the diagnosis of zoonotic disease is delayed through lack of clinical suspicion or failure to obtain adequate clinical histories. Some zoonotic infections are unusual (e.g., scabies infection after handling of pet guinea pigs). Other infections may have a less obvious animal link. Mowing lawns is believed to be a risk factor for acquiring tularemia (caused by *Francisella tularensis)* in disease-endemic areas where lagomorph reservoirs may be killed by mowers or hedge trimmers (*17*).

For some infections, zoonotic transmission occurs indirectly through food. Human brucellosis is not usually acquired through animal contact but is transmitted more often by consumption of infected animal products such as unpasteurized dairy products (*18*). *Salmonella* spp. have repeatedly caused outbreaks of salmonellosis after persons have eaten uncooked eggs (*19*). Hepatitis E virus has been transmitted through consumption of uncooked deer meat (*20*).

Exposure routes may be airborne, as demonstrated for several outbreaks of Q fever (*21*). An ongoing airborne Q fever outbreak in the Netherlands related to goat farming has raised awareness of this previously neglected zoonosis (*22*). How humans were exposed to these animals would not have been apparent; the exposures were identified by epidemiologic mapping of the distribution of cases. These examples underscore the necessity of gathering comprehensive patient data to effectively diagnose zoonoses.

## Recreational Zoonoses

Sporting activities can expose humans to zoonotic infections. Hunting wildlife has been associated with infections such as brucellosis and tularemia (*23*). Less obvious routes arise from activities such as water sports. *Leptospira* spp.–infected animals excrete viable organisms in their urine, which can persist in aquatic environments for prolonged periods. After a triathlon event in 1998, a total of 52 of 474 athletes tested were diagnosed with leptospirosis (*24*). Suspicion of water sport–related infections with hepatitis A and *Leptospira* spp. led to closure of an area of Bristol, United Kindom, where docks were used for recreational water activities (*25*).

Horses are now moved from countries in Europe to warmer regions (e.g., United Arab Emirates) to prolong the racing season during the winter. Hunting activities have promoted large-scale export of animals such as hares (possible reservoirs of tularemia and brucellosis) from Poland and the movement of potentially rabies-infected raccoons in the United States. In other countries such as the United Kingdom, pheasants are bred and released for shooting in the fall and provide plentiful hosts for questing ticks and increasing their abundance. Importation of pheasants into the United Kingdom from France was associated with introduction of a mild zoonotic infection (Newcastle virus disease) in 2007 (*26*).

## Role of Companion Animals

Companion animals have many forms of contact and opportunities to transmit multiple zoonoses. The sexual stage of the life cycle of *Toxplasma* spp. occurs in cats, thus exposing humans to infection in situations in which hygienic measures have not been observed. Cats also serve as reservoir for *Bartonella henselae*, the etiologic agent of cat-scratch fever (*27*). Cowpox virus can also be transmitted to humans by contact with cats (*28*). Animal bites can result in zoonotic infections, typified by infection with *Pasteurella multocida*. Even in the absence of a bite, contact with animals (e.g., licking of wounds) can result in infection. More recently, attention has focused on transmission of *Rickettsia felis* into the human environment by cat fleas (*29*).

Dogs are the most likely source when humans become infected with rabies virus and are potential sources of *Toxocara* spp. This emerging threat is becoming apparent with importation of rescued dogs and global movement of dogs with their owners, which has resulted in several cases of leishmaniasis in the absence of sand fly vectors. Dogs can be a source of methicillin-resistant *Staphylococcus aureus* and could play a role in zoonotic spread of genetic elements responsible for antimicrobial drug resistance (*30*). Contact with dogs in Mediterranean regions has been implicated as a likely source of infection in recent cases of Mediterranean spotted fever reported in traveling humans (*31*).

Cats and dogs can introduce plague or rabies into human environments and have been associated with Q fever in humans and dermatophytosis (ringworm). Scavenger habits of these animals bring them into contact with many zoonotic agents, and close living relationships with humans such as sharing meal plates or beds offer many opportunities for disease transmission.

Pet rats have recently been incriminated as the source of *Leptospira icterrohemoragiae* infection in their owners. Psittacine birds are an established risk factor for acquisition of *Chlamydophila psittaci*. During recent years, the market for exotic pets has greatly increased. This increase has resulted in transmission of several unusual organisms, such as exotic *Salmonella* spp., which are often associated with pet reptiles. Media attention was captured after an outbreak of monkeypox in America that affected >70 persons in 2003. After infected African rodents had been imported for the pet trade, the infection spread into native North American black-tailed prairie dogs and was subsequently disseminated among humans (*32*).

## Bush Meat

Zoonotic diseases associated with hunting and eating wildlife is of increasing global concern. Bush meat is considered a delicacy by many and has resulted in its growth as a commercial enterprise. Tracking, capturing, handling, butchering in the field, and transporting of carcasses involve risks of cross-species transmission. Particularly high risks are associated with hunting nonhuman primates. The act of butchering is a greater risk factor for acquiring bloodborne pathogens than transporting, selling, and eating the butchered meat (*33*).

Zoonotic pathogens from wildlife may infect humans with little or no human-to-human transmission (e.g., avian influenza virus and Hendra virus). Alternatively, increased travel or migration and increased between-person contacts have facilitated emergence of simian immunodeficiency virus/HIV/AIDS in Africa. Increased exposure to wild-caught animals and high mutation rates of many RNA viruses have increased their predominance among emerging zoonoses transmitted from human to human; RNA viruses from bush meat may therefore play a possible role in future disease emergence.

## Globalization and Livestock Movement

Large-scale movement of persons, livestock, food, or goods is now commonplace and provides increasing opportunities for rapid spread of pathogens. Trichinellae in horsemeat have been transported across the Pacific Ocean and infected consumers in other parts of the world. Discarded tires provide new habitats for mosquitoes in addition to their usual ecologic niches. The World Organisation for Animal Health and the Food and Agriculture Organisation implement strict control of animal movement. Transport of animals can result in mingling of different species in crowded and stressful conditions. This mingling can suppress immune responses to persistent infections and increase pathogen shedding. Under such circumstances, susceptible species can readily become infected (*34*).

## Tourism

Tourism has exponentially increased in recent years. This finding has resulted in increasing numbers of imported zoonoses, such as a variety of rickettsial spotted fevers, brucellosis, melioidosis, genotype I hepatitis E (*35*), tick-borne encephalitis (*36*), and schistosomiasis (*37*).A rapid increase in cases of African tick bite fever has been associated with travelers to sub-Saharan Africa and the eastern Caribbean. This disease, which is caused by *R*. *africae*, is transmitted by a particularly aggressive *Amblyomma* sp. tick; >350 imported cases have been observed in recent years (*31*). Infection sequalae, such as subacute neuropathy, may be found long after travel when tick bite fever eschars have disappeared (*37*). An estimated >1 million international journeys are made each day, and a staggering 700 million tourists travel on an annual basis. Detailed travel histories of patients who show clinical signs and symptoms of disease are needed.

## Changed Land Use and Urbanization

Deforestation and development of natural habitats have been seen on a global scale to accommodate intensification of agriculture and living areas for humans. As a result, ecologic habitats have been disrupted, reservoir abundance has changed, and transmission dynamics have been altered. Reduced host abundance may force vectors to seek alternative hosts, increasing opportunities for disease transmission, as demonstrated by increases in human cases of Lyme borreliosis, ehrlichiosis, spotted fevers, and anaplasmosis. Development of forests to provide rubber plantations in Malaysia has been correlated with increases in schistosomiasis (*37*). Wildlife may modify feeding practices as a consequence of changing land use, bringing them closer to humans and livestock. This modification was suggested to have been instrumental in the Nipah virus outbreak that affected pigs and humans in Malaysia in 1999. Nipah virus persists as a serious problem in many rural areas of Bangladesh and India, where infected bats living near human dwellings, urinate in date palm sap, which is later consumed raw by humans (*38*).

Human population growth has been associated with reshaping of population demographics. Increasing from 1 billion at the beginning of the 20th century to 6 billion by the end of the century, current predictions forecast a human population of ≈10 billion by 2050. This prediction is accompanied by a staggering increase in urbanization of the population from 39% in urban environments in 1980 to 46% in 1997 and a predicted 60% by 2030. This high-density clustering of the human population paves the way for potential outbreaks on an immense scale (*5*).

## Public Health Risks of Reemerging and Neglected Zoonoses

Many areas are now experiencing a reemergence of zoonotic pathogens, partly resulting from collapse of public health programs during political upheavals. Often, these areas increasingly appeal to those seeking adventurous or unusual holiday destinations.

Delay in development of clinical signs and often insidious onset can challenge appropriate diagnosis and patient management. Furthermore, movements of animals used for agricultural trade, sport, and as companions also offer opportunities for further dissemination of infections. Brucellosis-free countries have seen reintroductions associated with movement of infected livestock. Movement of pets throughout Europe has been associated with an alarming increase in diseases such as leishmaniasis. Moreover, pets can harbor ectoparasites such as ticks, fleas, and lice. All of these parasites, especially ticks, are notorious vectors of multiple zoonotic agents.

We are at risk for airborne transmission of zoonoses by many factors (e.g., from travel to farms, consumption of food, and mowing the lawn, which as been associated with tularemia). Visiting petting farms or having family pets increases the likelihood of potential zoonotic infections, especially if pets are exotic. Water sports may increase the risk for acquiring leptospirosis. Wilderness camping activities have been associated with hantavirus infection after inhalation of aerosolized urine excretions of rodents. Other sporting activities such as hunting have been associated with brucellosis and tularemia. Travel to other countries opens a range of new potential zoonotic exposures through direct contact or indirectly through fomites, food, or arthropod vectors. Increasingly exotic locations are being sought with associated exotic zoonoses. Some tourists consume local delicacies, such as aborted animal fetuses in Ecuador, which are a source of brucellosis (*39*).

## Conclusions and Future Prospects

Many zoonoses can be considered opportunistic infections. Increasing demands for protein necessitate increased levels of farming. Food can provide a vehicle for spread of pathogens from animals to humans. Contact with animals during farming, hunting, or by animal bites can increase transmission of diseases (e.g., rabies and tularemia). Arthropod vectors can transmit diseases on an immense scale to other hosts as in cases of West Nile fever and plague.

Changing patterns of farming, life style, and transportation influence the dynamics of pathogen ecology. Pathogens are subjected to changes by many intrinsic and extrinsic factors. Mutation, recombination, selection, and deliberate manipulation can result in new traits acquired by pathogens and result in potential epidemic consequences.

Reemergence of diseases through opportunistic host switching is likely to continue as a major source of human infectious disease. Strategies to improve public health have focused on improved surveillance in regions of perceived high likelihood of disease (reemergence). These strategies include improved detection of pathogens in reservoirs, early outbreak detection, broad-based research to identify factors that favor reemergence, and effective control (i.e., quarantine and improved hygiene) (*40*).

To recognize and combat zoonotic diseases, the epidemiology of these infections must be understood. We need to identify pathogens, their vertebrate hosts, and their methods of transmission. Identification should include knowledge of spatiotemporal disease patterns and their changes over time. These features can be used to identify dynamic processes involved in pathogen transmission (Figure), which can be used to account for observed disease patterns and ultimately forecast spread and establishment into new areas.

**Figure:**
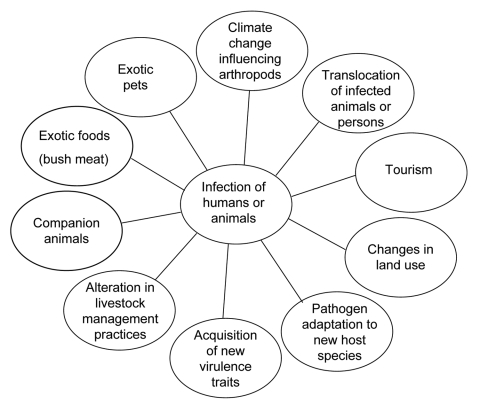
Factors influencing new and reemerging zoonoses.

Armed with information on expected disease patterns, we can address whether change has occurred beyond that which would normally be expected. However, this analysis may not be suitably responsive to control new and emerging zoonoses. Improved detection may be achieved through use of syndromic approaches rather than searching for specific pathogens.

Human disease surveillance must be associated with enhanced longitudinal veterinary surveillance in food-producing animals and wildlife. Prompt detection and instigation of control measures such as vaccination are pivotal to prevent disease spread. Novel molecular methods (e.g., DNA microarrays) offer unprecedented opportunities for rapid detection. However, these methods require optimization and validation before they can be used in routine microbiology laboratories. Cloned antigens or attenuated vaccines can be rapidly modified into appropriate antigenic forms. However, for identification of specific pathogens, more research will be needed to provide timely management of a new or emerging disease threat.

Approaches for identification of pathogen replication in vectors are more likely to offer substantial benefits for control of zoonoses. These methods are inappropriate for human vaccines, which must adhere to stricter legislative criteria. However, control of zoonotic infections in reservoir hosts has a pronounced protective effect in human populations. Use and development of antiviral drugs are other useful possibilities, but these drugs are likely to be too expensive for use in large disease outbreaks and emergence of drug resistance may result in concomitant loss of therapeutic options for these agents.

We do not know which zoonosis will be the next serious public health threat. However, as we increase efforts to improve the capacity to respond to this pathogen, we will also increase the likelihood that we can efficiently and effectively respond to new, reemerging, or neglected zoonoses in the future.

## References

[1] Daszak P, Epstein JH, Kilpatrick AM, Aguirre AA, Karesh WB, Cunningham AA. Collaborative research approaches to the role of wildlife in zoonotic disease emergence. Curr Top Microbiol Immunol. 2007;315:463–75. 10.1007/978-3-540-70962-6_18 17848075 PMC7122236

[2] Woolhouse ME, Gowtage-Sequeria S. Host range and emerging and re-emerging pathogens. Emerg Infect Dis. 2005;11:1842–7. 16485468 10.3201/eid1112.050997 PMC3367654

[3] Colizza V, Barrat A, Barthélemy M, Vespignani A. The role of the airline transportation network in the prediction and predictability of global epidemics. Proc Natl Acad Sci U S A. 2006;103:2015–20. 10.1073/pnas.0510525103 16461461 PMC1413717

[4] Fèvre EM, Bronsvoort BM, Hamilton KA, Cleaveland S. Animal movements and the spread of infectious diseases. Trends Microbiol. 2006;14:125–31. 10.1016/j.tim.2006.01.004 16460942 PMC7119069

[5] Jones KE, Patel NG, Levy MA, Storeygard A, Balk D, Gittleman JL, Global trends in emerging infectious diseases. Nature. 2008;451:990–4. 10.1038/nature06536 18288193 PMC5960580

[6] Mahy BW, Brown CC. Emerging zoonoses: crossing the species barrier. Rev Sci Tech. 2000;19:33–40. 11189724 10.20506/rst.19.1.1212

[7] Stavrinides J, Guttman DS. Mosaic evolution of the severe acute respiratory syndrome coronavirus. J Virol. 2004;78:76–82. 10.1128/JVI.78.1.76-82.2004 14671089 PMC303383

[8] Haydon DT, Cleaveland S, Taylor LH, Laurenson MK. Identifying reservoirs of infection: a conceptual and practical challenge. Emerg Infect Dis. 2002;8:1468–73. 12498665 10.3201/eid0812.010317 PMC2738515

[9] Outbreak news. Plague, Democratic Republic of The Congo. Wkly Epidemiol Rec. 2006;81:397–8. 17068850

[10] Girard JM, Wagner DM, Vogler AJ, Keys C, Allender CJ, Drickamer LC, Differential plague-transmission dynamics determine *Yersinia pestis* population genetic structure on local, regional, and global scales. Proc Natl Acad Sci U S A. 2004;101:8408–13. 10.1073/pnas.0401561101 15173603 PMC420407

[11] Bengis RG, Leighton FA, Fischer JR, Artois M, Mörner T, Tate CM. The role of wildlife in emerging and re-emerging zoonoses. Rev Sci Tech. 2004;23:497–511. 15702716

[12] Galimand M, Carniel E, Courvalin P. Resistance of *Yersinia pestis* to antimicrobial agents. Antimicrob Agents Chemother. 2006;50:3233–6. 10.1128/AAC.00306-06 17005799 PMC1610074

[13] Michel H, Wilske B, Hettche G, Göttner G, Heimerl C, Reischl U, An ospA-polymerase chain reaction/restriction fragment length polymorphism-based method for sensitive detection and reliable differentiation of all European *Borrelia burgdorferi* sensu lato species and OspA types. Med Microbiol Immunol (Berl). 2004;193:219–26. 10.1007/s00430-003-0196-8 13680214

[14] Duffy S, Shackleton LA, Holmes EC. Rates of evolutionary change in viruses: patterns and determinants. Nat Rev Genet. 2008;9:267–76. 10.1038/nrg2323 18319742

[15] Parrish CR, Holmes EC, Morens DM, Park EC, Burke CH, Laughlin CA, Cross-species virus transmission and the emergence of epidemic diseases. Microbiol Mol Biol Rev. 2008;72:457–70. 10.1128/MMBR.00004-08 18772285 PMC2546865

[16] Anishchenko M, Bowen RA, Paessler S, Austgen L, Greene IP, Weaver SC. Venezuelan encephalitis emergence mediated by a phylogenetically predicted viral mutation. Proc Natl Acad Sci U S A. 2006;103:4994–9. 10.1073/pnas.0509961103 16549790 PMC1458783

[17] Agger WA, Goethert HK, Telford SR III. Tularemia, lawn mowers, and rabbits’ nests. J Clin Microbiol. 2005;43:4304–5. 10.1128/JCM.43.8.4304-4305.2005 16082010 PMC1233993

[18] Chomel BB, DeBess EE, Mangiamele DM, Reilly KF, Farver TB, Sun RK, Changing trends in the epidemiology of human brucellosis in California from 1973 to 1992: a shift toward foodborne transmission. J Infect Dis. 1994;170:1216–23. 7963716 10.1093/infdis/170.5.1216

[19] Duguid JP, North RA. Eggs and *Salmonella* food-poisoning: an evaluation. J Med Microbiol. 1991;34:65–72. 10.1099/00222615-34-2-65 1990142

[20] Tei S, Kitajima N, Takahashi K, Mishiro S. Zoonotic transmission of hepatitis E virus from deer to human beings. Lancet. 2003;362:371–3. 10.1016/S0140-6736(03)14025-1 12907011

[21] Salmon MM, Howells B, Glencross EJ, Evans AD, Palmer SR. Q fever in an urban area. Lancet. 1982;1:1002–4. 10.1016/S0140-6736(82)92000-1 6122818

[22] Karagiannis I, Schimmer B, van Lier A, Timen A, Schneeberger P, van Rotterdam B, Investigation of a Q fever outbreak in a rural area of The Netherlands. Epidemiol Infect. 2009;137:1283–94. 10.1017/S0950268808001908 19161644

[23] Bourque M, Higgins R. Serologic studies on brucellosis, leptospirosis and tularemia in moose (*Alces alces*) in Quebec. J Wildl Dis. 1984;20:95–9. 6429355 10.7589/0090-3558-20.2.95

[24] Morgan J, Bornstein SL, Karpati AM, Bruce M, Bolin CA, Austin CC, Outbreak of leptospirosis among triathlon participants and community residents in Springfield, Illinois, 1998. Clin Infect Dis. 2002;34:1593–9. 10.1086/340615 12032894

[25] Philipp R, Waitkins S, Caul O, Roome A, McMahon S, Enticott R. Leptospiral and hepatitis A antibodies amongst windsurfers and waterskiers in Bristol city docks. Public Health. 1989;103:123–9. 10.1016/S0033-3506(89)80026-5 2786228

[26] Aldous EW, Manvell RJ, Cox WJ, Ceeraz V, Harwood DG, Shell W, Outbreak of Newcastle disease in pheasants (*Phasianus colchicus*) in south-east England in July 2005. Vet Rec. 2007;160:482–4. 17416725 10.1136/vr.160.14.482

[27] Arvand M, Schad SG. Isolation of *Bartonella henselae* DNA from the peripheral blood of a patient with cat scratch disease up to 4 months after the cat scratch Injury. J Clin Microbiol. 2006;44:2288–90. 10.1128/JCM.00239-06 16757642 PMC1489392

[28] Haenssle HA, Kiessling J, Kempf VA, Fuchs T, Neumann C, Emmert S. Orthopoxvirus infection transmitted by a domestic cat. J Am Acad Dermatol. 2006;54:S1–4. 10.1016/j.jaad.2005.09.040 16427982

[29] Gilles J, Just FT, Silaghi C, Pradel I, Lengauer H, Hellmann K, *Rickettsia felis* in fleas, France. Emerg Infect Dis. 2008;14:684–6. 10.3201/eid1404.071103 18394302 PMC2570947

[30] Epstein CR, Yam WC, Peiris JSM, Epstein RJ. Methicillin-resistant commensal staphylococci in healthy dogs as a potential zoonotic reservoir for community-acquired antibiotic resistance. Infect Genet Evol. 2009;9:283–5. 10.1016/j.meegid.2008.11.003 19073283

[31] Jensenius M, Fournier PE, Raoult D. Rickettsioses and the international traveler. Clin Infect Dis. 2004;39:1493–9. 10.1086/425365 15546086

[32] Hutson CL, Lee KN, Abel J, Carroll DS, Montgomery JM, Olson VA, Monkeypox zoonotic associations: insights from laboratory evaluation of animals associated with the multi-state US outbreak. Am J Trop Med Hyg. 2007;76:757–68. 17426184

[33] Wolfe ND, Daszak P, Kilpatrick AM, Burke DS. Bushmeat hunting, deforestation, and prediction of zoonotic disease emergence. Emerg Infect Dis. 2005;11:1822–7. 16485465 10.3201/eid1112.040789 PMC3367616

[34] Stark JH, Basetse HR, Lecatsas G, Smit JA, Myburgh JA. Xenozoonoses: assessing activation of latent/unknown viruses in immunosuppressed baboons. Transplant Proc. 1996;28:856–7. 8623435

[35] Dalton HR, Thurairajah PH, Fellows HJ, Hussaini HS, Mitchell J, Bendall R, Autochthonous hepatitis E in southwest England. J Viral Hepat. 2007;14:304–9. 10.1111/j.1365-2893.2006.00800.x 17439519

[36] Jensenius M, Parola P, Raoult D. Threats to international travellers posed by tick-borne diseases. Travel Med Infect Dis. 2006;4:4–13. 10.1016/j.tmaid.2004.11.003 16887719

[37] Meltzer E, Artom G, Marva E, Assous MV, Rahav G, Schwartzt E. Schistosomiasis among travelers: new aspects of an old disease. Emerg Infect Dis. 2006;12:1696–700. 17283619 10.3201/eid1211.060340 PMC3372337

[38] Luby SP, Rahman M, Hossain MJ, Blum LS, Husain MM, Gurley E, Foodborne transmission of Nipah virus, Bangladesh. Emerg Infect Dis. 2006;12:1888–94. 17326940 10.3201/eid1212.060732 PMC3291367

[39] Godfroid J, Cloeckaert A, Liautard J, Kohler S, Fretin D, Walravens K, From the discovery of the Malta fever’s agent to the discovery of a marine mammal reservoir, brucellosis has continuously been a re-emerging zoonosis. Vet Res. 2005;36:313–26. 10.1051/vetres:2005003 15845228

[40] Fraser C, Riley R, Anderson RM, Ferguson NM. Factors that make an infectious disease outbreak contollable. Proc Natl Acad Sci U S A. 2004;101:6146–51. 10.1073/pnas.0307506101 15071187 PMC395937

